# Clinical evaluation of atypical glandular cells of undetermined significance upon cervical cytologic examination in Israeli Jewish women

**DOI:** 10.1038/sj.bjc.6601874

**Published:** 2004-05-11

**Authors:** G Gutman, R Bachar, D Pauzner, J B Lessing, E Schejter

**Affiliations:** 1 Colposcopic Clinic, Maccabi Health Services, Tel Aviv, Israel; 2 LIS Maternity Hospital, Tel Aviv Sourasky Medical Center affiliated to the Sackler Faculty of Medicine, Tel Aviv University, Tel Aviv, Israel

**Keywords:** AGUS, ASCUS, bethesda system, cervical intraepithelial neoplasia

## Abstract

The adjusted incidence of cervical carcinoma among Israeli Jewish women is ∼5 out of 100 000. This retrospective study sought to determine the clinical implications of finding atypical glandular cells of undetermined significance (AGUS) in cervical cytologic specimens in this population. Cervical cytologic examinations during January 2001–June 2003 diagnosed as AGUS were identified by a computerised database. Medical records were reviewed to determine the presence or absence of associated significant pathologic conditions of the cervix and identified 45 out of 11 800 patients (0.38%) with AGUS. AGUS was the only cytologic diagnosis in 14 patients, while 31 patients had both AGUS and an additional atypical squamous cell of undetermined significance (ASCUS). All subjects underwent colposcopy, endocervical curettage, and cervical biopsy. A clinically significant diagnosis (cervical intraepithelial neoplasia (CIN) II, CIN III, or carcinoma) was made in 24 patients (53.3%), including cancer in three (6.7%): one had microinvasive adenocarcinoma and two had microinvasive squamous cell carcinoma. Squamous carcinoma coexisting with a clinically significant lesion carried a risk of 61.3%, compared with a risk of 35.7% for AGUS alone (*P*=0.20). Detection of AGUS during cervical cytologic screening, especially with a coexisting ASCUS, indicates the existence of serious pathologic processes; management by cervical colposcopy, endocervical curettage, and cervical biopsy is recommended.

Israeli Jewish women are at low risk for cancer of the uterine cervix (Sadan *et al*, 2003). Cervical carcinoma ranks 20th among all their cancers, with the adjusted incidence rate being ∼5 out of 100 000, a figure among the lowest worldwide. The data recently published by Sadan *et al* (2003) now indicate similar incidence rates for premalignant lesions in Jewish Israeli women as observed in Western countries. The current study was undertaken to evaluate the efficacy of follow-up methods and the nature of the results of atypical glandular cells of undetermined significance (AGUS) detected on cervical Pap smears in this unique population.

The Bethesda System for cytologic reporting of Papanicolaou (Pap) smears was originally developed in 1988 (revised in 1991) in order to provide a standardised method of reporting cytologic findings for facilitating peer review and quality assurance. The 2001 version of the Bethesda System reflects the most current knowledge about the biology of Pap test abnormalities (Solomon *et al*, 2002).

In the current paper, we used the term ‘atypical cell’ to describe those cellular changes that are neither characterised by infection nor any preneoplastic, neoplastic, or reparative processes, and not to denote inflammatory or reactive cellular changes. The term AGUS was reserved for atypical cells in which a glandular origin was suspected, and the term ‘atypical squamous cells of undetermined significance’ (ASCUS) was reserved for atypical cells in which a squamous origin was suspected.

In contrast to both preinvasive and neoplastic squamous lesions of the cervix, where the evaluation and treatment are usually straightforward, glandular lesions of the cervix are diagnostically and therapeutically more challenging to the cytopathologist and clinician because of their relative rarity, the relative absence of colposcopic findings, irregular shedding, small size, endocervical location (making lesions less amenable to cytologic sampling), and broader differential diagnosis (Goff *et al*, 1992). Moreover, benign conditions such as cervical endometriosis, tubal metaplasia, a history of cone biopsy, Gartner's ducts, microglandular hyperplasia, deciduosis, and even endometrial glands in the upper cervical canal can make the differential diagnosis a difficult task (Pacey *et al*, 1988; Novotny *et al*, 1992; Lee, 1993).

The classification of glandular abnormalities was significantly revised in the 2001 Bethesda System, reflecting a reappraisal of the strengths and weaknesses of cytology in assessing these findings. According to that system, glandular cell abnormalities are classified as ‘atypical endocervical, endometrial, or glandular cells.’ In most cases, the morphological features of the condition enable differentiating between atypical endometrial and endocervical cells (Wilbur, 1997). The management of patients with glandular abnormalities may vary significantly, depending upon cell type, thus justifying making this distinction when possible.

## MATERIALS AND METHODS

This study was conducted over a period of 2 years and 6 months (from January 2001 to June 2003). During this period, 11 800 patients were examined by the Colposcopic Unit of the Balfour-Maccabi Women's Healthcare Center (Tel-Aviv, Israel). This unit is a public facility and is subsidised by the national governmental health insurance scheme. Two senior gynecologists who are specialists in cervical medicine performed the colposcopies (RB and ES).

Of these 11 800 samples, 45 (0.38%) Pap smears showed AGUS and comprised our study data. These 45 women were examined by colposcopic examination, colposcopic-directed biopsy, cone biopsy, and/or endocervical curettage.

The medical records of all AGUS patients were reviewed and the retrieved data consisted of the woman's age, gravidity, parity, menopausal status, as well as any history of abnormal bleeding, smoking, use of oral contraceptives, intrauterine devices, replacement of hormones, prior abnormal Pap smear, or previous cancer. In addition, the results of the clinical evaluation of their AGUS smear were collected, including the findings of the physical examination, colposcopy, cytology, and biopsy results. Cervical biopsies were classified according to the modified Richart classification system for cervical intraepithelial neoplasia (CIN), where lesions consistent with flat condyloma and CIN I are referred to as ‘low-grade CIN’ and lesions consistent with CIN II or CIN III are grouped together as ‘high-grade CIN’ (Richart, 1990). The AGUS pap smears in this study were reviewed by a single cytopathologist. For the purposes of this study, a ‘clinically significant lesion’ was defined as CIN II, CIN III, or carcinoma. CIN I was not considered a clinically significant lesion and was therefore classified as benign.

## RESULTS

There were 45 patients (mean age 41.3 years) in the study cohort. The smears of 14 of them (31%) were reported as due to AGUS alone and 31 (69%) smears had coexistent squamous pathology. Seven (16%) patients had a recent history of abnormal vaginal or postcoital bleeding. Four patients had a history of nongynecologic cancer: three had breast cancer and one had carcinoma of the rectum. One of the patients was pregnant at the time of her AGUS cytology finding.

All 45 patients were colposcoped and biopsied shortly after AGUS was confirmed: 16 women (36%) underwent colposcopy with cervical biopsies, 29 (64%) underwent colposcopy with cervical biopsies and endocervical curettage, and 25 (55%) of the women who underwent cervical biopsies required diagnostic conisation during their workup. Since our protocol dictates that women with AGUS and without clear external lesions undergo endocervical curettage, the 16 women who had clear external lesions did not undergo this procedure.

Table 1

**Table 1:** Histopathology in the study women with AGUS smears (*n*=45)

**Diagnosis**	***n* (%)**
Normal	10 (22)
Cervicitis	4 (9)
CIN I	7 (15)
CIN II–III	21 (47)
Carcinoma	3 (7)

CIN=cervical intraepithelial neoplasia; AGUS=atypical glandular cells of uncertain significance.

shows the pathology identified in the smears of the study group. Table 2

**Table 2:** Histopathology in women with AGUS smears with and without coexistent squamous abnormalities

**Diagnosis**	**AGUS alone (*n*=14)**	**AGUS+ASCUS (*n*=31)**
Normal	4	6
Cervicitis	2	2
CIN I	3	4
CIN II–III	4	17
Carcinoma	1	2

a *P*=NS between the two groups. ASCUS=atypical squamous cell of undetermined significance; CIN=cervical intraepithelial neoplasia; AGUS=atypical glandular cells of uncertain significance.

compares the pathology found in women with AGUS with and without concomitant squamous abnormalities. A patient diagnosed with coexistent ASCUS carried a risk factor of 61.3% for the existence of a clinically significant lesion, compared with a risk of 35.7% for AGUS alone. When Fischer's exact test was used to compare these groups, however, the women with coexistent squamous abnormalities detected by a Pap smear were not found to have significantly more cervical lesions than women with AGUS alone (*P*>0.05). Only one of the 45 patients with AGUS was found to have adenocarcinoma of the cervix.

In total, 14 (31%) of the study patients were postmenopausal, and our results indicated that both premenopausal and postmenopausal patients were at risk for clinically significant lesions. Moreover, there was no difference between pre- and postmenopausal women in terms of pathology. Even so, endometrial biopsy is recommended in postmenopausal women with Pap-diagnosed AGUS who do not have an external lesion on cervical or endocervical biopsy. All of our postmenopausal study women had significant cervical lesions and did not require endometrial biopsy.

## DISCUSSION

Glezerman *et al* (1989) noted a remarkable, although not statistically significant, increase in the number of Israeli Jewish women patients with cervical cancer. Sadan *et al* (2003) retrospectively analysed the results of 297 849 Pap smears during 9 years (1991–1999), and their data indicated similar incidence rates for premalignant lesions in Jewish Israeli women as observed in Western countries. Bar-Am *et al* (1995) found that the prevalence of abnormal cytology among the Israeli women was almost the same as that of a non-Jewish group (24 out of 1000 and 26 out of 1000, respectively), and that the distribution of the known risk factors among the study group (number of sexual partners, age at first intercourse, and age at first pregnancy) was practically the same as those in the non-Jewish population. The authors documented an increase of 29.2% (from 17 out of 1000 to 24 out of 1000) in the prevalence of cervical premalignant lesions among the Israeli women in their study group and noted that they were younger when they became sexually active, had a larger number of sexual partners, and fewer of them were married. In addition, fewer of them were observant of Jewish ritual practices, one of which is abstinence from intercourse during menstruation and for 1 week afterwards. We designed the current study to document whether this growing similarity between Jewish Israeli and other groups of women was also reflected by the pattern of AGUS in their Pap smears and our findings showed that here, too, this population no longer enjoys a comparatively lower risk.

The optimal clinical evaluation of the diagnosis of AGUS by the Pap test has yet to be determined. Therefore, the clinician is faced with uncertainty as to the aggressiveness of the abnormal cells and the proper sequence of clinical evaluation. Some physicians choose a conservative approach to AGUS and treat it much like ASCUS, with repeated cytologic examination alone, whereas others take a more comprehensive approach, performing colposcopy and biopsy of the endocervix.

The diagnosis of AGUS is made in routine clinical practice in 0.13–2.5% of the smears; most rates quoted in the literature are less than 1% (Goff *et al*, 1992; Nasu *et al*, 1993; Lee *et al*, 1995; Ferris *et al*, 1996; Kennedy *et al*, 1996; Raab *et al*, 1997; Zweizig *et al*, 1997). Thus, our diagnostic rate of 0.38% in this study is in agreement with the current literature.

The present study reviewed a series of 45 patients with AGUS as diagnosed by a cytologic screening exam. In previous studies, as well as this one, AGUS has been associated with significant histologic abnormalities in 53.3% of all cases, including cancer in 6.7% (Richart, 1990; Goff *et al*, 1992; Eddy *et al*, 1997; Zweizig *et al*, 1997; Veljovich *et al*, 1998). With one exception, all of these abnormalities were confirmed by cytologic evaluation as being due to AGUS and squamous in nature. In our study, the prevalence of significant cervical disease was equally distributed between premenopausal and postmenopausal cases.

The number of clinically significant lesions in the group we studied is sufficiently high to suggest that a complete evaluation (colposcopy with cervical biopsies, endocervical curettage, and diagnostic conisation when needed) of all women with an AGUS smear is warranted. An endometrial biopsy is warranted in the absence of persistent AGUS in the cervical findings from the biopsy (Zweizig *et al*, 1997).

We found significant squamous lesions in 51% of the AGUS smears and only 2.2% glandular lesions. The possible explanation for the relative absence of glandular pathology is the fact that the cells were so badly deformed that they appeared to be glandular to the cytopathologist but were actually squamous in origin.

Finally, the results of this study strongly support that the presence of AGUS cells upon a routine cytologic specimen examination indicates the existence of a pathologic condition that mandates appropriate management.
